# Physical Concept to Explain the Regulation of Lipid Membrane Phase Separation under Isothermal Conditions

**DOI:** 10.3390/life13051105

**Published:** 2023-04-28

**Authors:** Naofumi Shimokawa, Tsutomu Hamada

**Affiliations:** School of Materials Science, Japan Advanced Institute of Science and Technology, 1-1 Asahidai, Nomi 923-1292, Ishikawa, Japan

**Keywords:** phase separation, lipid membrane, giant vesicle, liposome, free energy, charged lipid, photoisomerization, membrane tension, undulation

## Abstract

Lateral phase separation within lipid bilayer membranes has attracted considerable attention in the fields of biophysics and cell biology. Living cells organize laterally segregated compartments, such as raft domains in an ordered phase, and regulate their dynamic structures under isothermal conditions to promote cellular functions. Model membrane systems with minimum components are powerful tools for investigating the basic phenomena of membrane phase separation. With the use of such model systems, several physicochemical characteristics of phase separation have been revealed. This review focuses on the isothermal triggering of membrane phase separation from a physical point of view. We consider the free energy of the membrane that describes lateral phase separation and explain the experimental results of model membranes to regulate domain formation under isothermal conditions. Three possible regulation factors are discussed: electrostatic interactions, chemical reactions and membrane tension. These findings may contribute to a better understanding of membrane lateral organization within living cells that function under isothermal conditions and could be useful for the development of artificial cell engineering.

## 1. Introduction

Fluid bilayer membranes that mainly consist of lipids are common boundaries that enclose living cells and cellular organelles, such as the endoplasmic reticulum and Golgi apparatus [1]. Dynamic organization of the lipid bilayer membrane is essential for cellular functions. Since biological membranes are self-assembling structures with many lipid molecules (~10^7^ lipids form a membrane surface of several μm^2^), they show thermodynamic behaviors as two-dimensional fluids. One of these physical phenomena is lateral phase separation. Biological membranes with a multi-component lipid mixture are laterally segregated to form immiscible domains with high lipid order, called lipid rafts [2]. These raft domains are considered to be a form of liquid–liquid phase separation, i.e., liquid-ordered (L_o_) and liquid-disordered (L_d_) phases coexist [3,4]. The segregated lateral domains in biomembranes are proposed to serve as a subcompartment to support the localization of specific molecules for signal transduction [5]. The membrane domain could be related to human health, such as with regard to viral infections, cancer and Alzheimer’s disease [6,7]. Recently, in addition to plasma membrane, intracellular membranes, such as endoplasmic reticulum (ER), have been reported to show phase separation [8,9]. Much attention has been given to the mechanism of the regulation of lateral phase separation within lipid bilayer membranes in the fields of not only cell biology but also biophysics.

**Figure 1:**
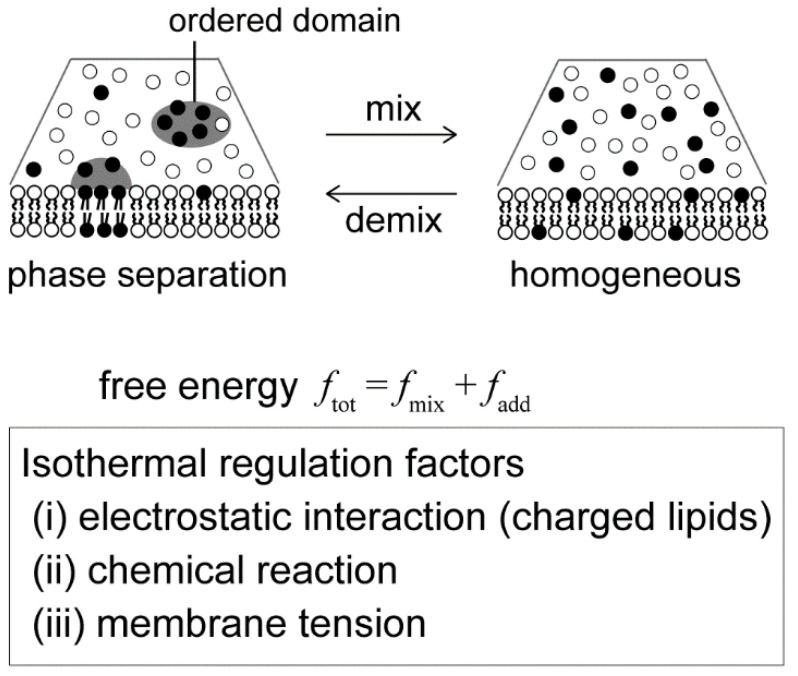
Isothermal regulation of membrane phase separation. We discuss physical mechanisms in terms of the free energy of the membrane as *f*_tot_ = *f*_mix_ + *f*_add_, where *f*_mix_ is the conventional Flory–Huggins theory, as discussed in Section 2. The effect of electrostatic interaction (*f*_add_ = *f*_el_) is described in Section 3. A chemical reaction (*f*_add_ = *f*_ch_) is considered in Section 4. The membrane undulation term (*f*_add_ = *f*_und_) is shown in Section 5.

Recently, artificial model membrane systems, called cell-sized liposomes or giant unilallelar vesicles (GUVs), have been attracting attention in studies of the physical principles that govern dynamical behaviors of the membrane [10,11,12,13]. Model membranes can be prepared to mimic cell membrane structures in terms of size (with a diameter of >10 μm) and membrane composition (multi-component systems). The structures and dynamics of the model membranes can be observed using optical microscopy [14,15]. With the use of model membranes, lateral phase separation has been visualized in a membrane surface consisting of a ternary lipid mixture, such as an unsaturated lipid with a low melting temperature, a saturated lipid with a high melting temperature and cholesterol (Figure 1) [16,17,18]. The localization of various molecules in phase-separated domains has been studied [19,20]. It is well known that phase separation is a thermodynamic behavior and depends on temperature [21]. Basically, the competition between the positional mixing entropy (*S*) and interaction energy among molecules (*U*) determines the stability of phase separation, i.e., the minimization of free energy *F* = *U* − *TS*. Below the miscibility temperature, phase separation occurs, and domains are formed in a system. At a high temperature, phase separation disappears to give a homogeneous state due to mixing entropy. Along these lines, there have been many studies with GUVs on membrane phase diagrams with changes in temperature [22,23,24,25]. However, living systems function under isothermal conditions, and thus cells should regulate phase-separated membrane domains without changes in temperature. Therefore, we should consider other physicochemical parameters to understand possible factors that regulate membrane phase behaviors in living cells.

In this review, we describe the regulation of phase separation under isothermal conditions using model membrane systems and explain physical mechanisms in terms of free energy. First, we introduce the free energy of the membrane based on the Flory–Huggins theory. Next, we consider additional factors/terms that contribute to the regulation of phase separation under isothermal biological conditions: (i) We expand the theory to include lipid charge, which is an intrinsic property of the membrane, and explain the mechanism of phase separation in charged membranes. (ii) Next, we explain the emergence and disappearance of domains by chemical reaction within the membrane. (iii) Finally, we focus on a mesoscopic fluid property of the membrane interface and show that osmotic tension enhances the stability of membrane phase separation. Notably, we do not discuss large deformations of membrane morphology. The lipid bilayer has a bending modulus of *κ*~20–30 *k*_B_*T*, and phase-separated membranes are known to tend to exhibit budding shapes [18,26].

## 2. Free Energy of Phase Separation

In this section, we introduce theoretical models for describing phase separation in multi-component membranes. Based on a thermodynamic approach, the equilibrium phase and structure are determined by minimizing the free energy. First, we use the Flory–Huggins theory to consider macro-phase separations in a neutral membrane. The lateral organization, known as the raft model, is regarded as phase separation in lipid membranes. In most cases, phase-separated domains are coalesced into a monodomain on the order of μm in size. The Flory–Huggins theory was originally proposed to explain phase separation in a polymer solution [27,28]. However, this model can also be used for qualitative analysis in phase-separated membranes.

For simplicity, we consider a binary monolayer membrane consisting of two neutral lipids with the same cross-sectional area ∑. Although Wagner and coworkers proposed the Flory–Huggins theory for bilayer membranes, we do not consider the bilayer nature [29]. Since the membrane is large enough, we ignore the membrane thickness. Moreover, the membrane is assumed to be a flat and undeformable elastic sheet. The mole fraction of one lipid is denoted by *ϕ*_n_ (0 < *ϕ*_n_ < 1) and the free energy per lipid molecule *f*_mix_ is given by the following:

(1) $$f_{\mathrm{mix}}(\phi_{n})=\phi_{n}\ln\phi_{n}+(1-\phi_{n})\ln(1-\phi_{n})+\chi \phi_{n}(1-\phi_{n})$$

where the energy is measured in units of *k*_B_*T* (*k*_B_ is the Boltzmann constant and *T* is temperature) and *χ* is a parameter that reflects repulsion between two types of lipid. The first and second terms indicate the mixing entropy of lipids, and the last term represents the interaction between two lipids that enhances lipid–lipid demixing. Generally, *χ* is proportional to the inverse of the temperature *χ*~1/*T*. The chemical potential *μ*_n_ of one lipid is calculated from *μ*_n_ = ∂*f*_mix_/∂*ϕ*_n_ and can be expressed as follows:

(2) $$\mu_{n}=\ln\frac{\phi_{n}}{1-\phi_{n}}+\chi (1-2\phi_{n})$$

The thermodynamic potential is defined by *g*_n_ ≡ *f*_mix_ − *μ*_n_*ϕ*_n_ and the coexistence between phases A and B is determined by *g*_nA_ = *g*_nB_ and *μ*_nA_ = *μ*_nB_. In other words, the two coexisting phases are obtained by drawing a common tangent line to the free energy. The spinodal line is obtained from the condition ∂^2^*f*_mix_/∂*ϕ*_n_^2^ = 0 as follows:

(3) $$\chi_{\mathrm{sp}\_n}=\frac{1}{2\phi_{n}(1-\phi_{n})}$$

**Figure 2:**
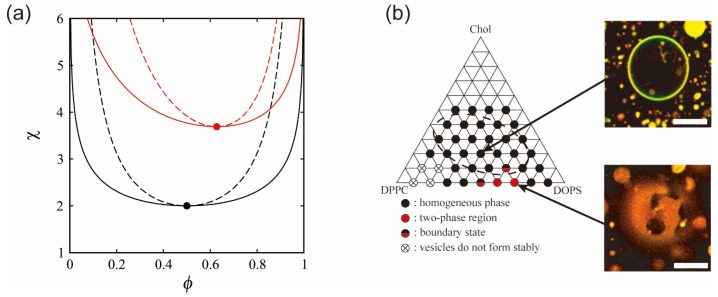
(**a**) Phase diagrams as a function of the lipid mole fraction *ϕ* and the interaction strength *χ* in neutral (black line) and charged (red line) membranes. *ϕ* represents *ϕ*_n_ in the neutral membrane and *ϕ*_c_ in the charged membrane (negatively charged lipid mole fraction). The solid and dashed lines indicate the binodal and spinodal lines, respectively. The filled circles denote the critical points located at (*ϕ*_nc_, *χ*_nc_) = (0.5, 2) and (*ϕ*_cc_, *χ*_cc_) = (0.628, 3.69). The homogeneous phase corresponds to the region below the binodal line. (**b**) Experimentally obtained phase diagram of a ternary lipid mixture (charged DOPS/neutral DPPC/Chol) together with microscopic images of homogeneous and phase-separated vesicles. The dashed elliptical region is the approximate region where phase separation is observed for neutral lipid mixtures. Scale bars are 10 µm. Reprinted from Ref. [30], Copyright 2010 with permission from Elsevier.

The critical point is given by ∂*χ*_sp_n_/∂*ϕ*_n_ = 0, and is located at (*ϕ*_nc_, *χ*_nc_) = (0.5, 2). Hence, at *χ* > 2, the membrane is separated into two phases. The phase behavior of a neutral membrane is summarized as a phase diagram in the (*ϕ*_n_, *χ*) plane, as shown at the bottom of Figure 2a. The solid and dashed lines denote binodal and spinodal lines, respectively. The filled circle corresponds to the critical point. The binodal line divides the homogeneous phase from the two-phase coexistence region, and the homogeneous phase is below the binodal line. The region between the binodal and spinodal lines is the metastable region.

There are some inadequacies in explaining the phase separation of lipid membranes with only the Flory–Huggins theory [31]. The first point is that we ignore the phase transition of lipids. Dioleoyl phosphatidylcholine (DOPC) and dipalmitoyl phosphatidylcholine (DPPC) are typical zwitterionic phospholipids, and membranes composed of DOPC and DPPC usually show phase separation at room temperature. These lipids show a phase transition between the solid (gel) phase and liquid (liquid crystalline) phase. This phase transition is called the “main transition” or “chain melting transition”. The phase transition temperatures are −17 °C for DOPC and 42 °C for DPPC. In order to describe such a phase transition, it is necessary to consider the free energy associated with the lipid chain melting. By combining the lipid phase transition and the phase separation based on the Flory–Huggins theory, experimental phase diagrams were reproduced quantitatively [32,33]. Phase diagrams of lipid membranes with the rippled phase (where a membrane shape is spatially modulated [34]) or with the presence of charged lipids [35] have also been calculated.

The other important point is that we ignore the effect of cholesterol in these models. Cholesterol increases lipid fluidity when interacting with saturated lipids, such as DPPC. Specifically, the solid-ordered phase of DPPC is changed into the liquid-ordered phase [36]. This interaction is highly specific and is difficult to describe with a simple phenomenological model. Therefore, many models ignore cholesterol and treat lipids such as DPPC, which are normally in the solid phase, as being in the liquid phase, i.e., DPPC and cholesterol are treated as one component in a liquid phase. On the other hand, there are some challenging phenomenological models that theoretically describe the physical properties of cholesterol. Putzel and Schick considered the model for a neutral system consisting of a saturated lipid, unsaturated lipid and cholesterol [37]. They introduced an order parameter, which describes the order of the entire saturated chain. This model is similar to the regular solution theory which is consistent with the Flory–Huggins theory for a ternary system. We will discuss the Flory–Huggins theory for a ternary system in Section 4.

It should also be noted that the Flory–Huggins theory plays an important role in macro-phase separation. Several reports have described micro-phase separation in bilayer membranes [38,39,40,41]. Some reports have discussed micro-phase separation in lipid monolayer and bilayer membranes [42,43,44,45]. We consider here the mixing entropy of lipids under the Flory–Huggins theory for simplicity. As a model of the complexity of biomembranes, it has been reported that the entropy of membrane-bound polymers can affect membrane phase separation [46].

## 3. Phase Separation in Charged Membranes

Next, we consider the effects of an intrinsic property of membrane components, such as electrostatic interaction, on the free energy of phase separation. The manner of the assembly of charged biomolecules is affected by Coulombic forces. Biological membranes contain many negatively charged lipids, e.g., the mitochondrial membrane has several charged lipids, and there are also charged membrane-embedded proteins [1]. In addition, the electrostatic potential at the membrane surface is influenced by the salt concentration (cation concentration), such as sodium, potassium and calcium ions, in intracellular or extracellular solution. By adjusting the salt concentration in the solution, it is possible to control the phase separation structure without changing the temperature by changing the electrostatic interaction. Electrostatic interaction may play an important role in the lateral organization of membranes under isothermal conditions. The effects of charged lipids on phase separation have been discussed in several experimental studies using model membranes [30,47,48,49]. The phase separation in bilayer membranes consisting of a neutral saturated lipid, a negatively charged unsaturated lipid and cholesterol is suppressed because of electrostatic repulsion between the charged lipids (Figure 2b). Furthermore, as the added salt concentration is increased, the phase separation region becomes larger and the miscibility temperature increases. To explain the phase separation in charged membranes, May and coworkers considered the electrostatic interaction in several theoretical models based on the Flory–Huggins theory with the Poisson–Boltzmann theory [50,51,52]. Next, we introduce this model for phase separation in binary charged membranes.

We assume almost the same situation as in the case of a neutral membrane mentioned in Section 2. The binary monolayer membrane is regarded as an undeformable flat sheet. Here, we consider a membrane consisting of a neutral lipid and a negatively charged lipid with the same cross-sectional area ∑. Although models of charged bilayer membranes have also been calculated [53,54,55], we do not mention such models in this review. The membrane is placed in the *xy* plane. It is located at *z* = 0 and is in contact with a symmetric monovalent salt solution (e.g., NaCl) at *z* > 0. When *ϕ*_c_ is the mole fraction of the charged lipid, the free energy *f*_tot_ can be expressed as follows:

(4) $$f_{\mathrm{tot}}(\phi_{c})=f_{\mathrm{mix}}(\phi_{c})+f_{\mathrm{el}}(\phi_{c})$$

*f*_mix_ is obtained from Equation (1) and represents nonelectrostatic contributions. On the other hand, *f*_el_ includes electrostatic interactions. The electrostatic free energy is obtained through the charging process [56]:

(5) $$f_{\mathrm{el}}(\phi_{c})=\frac{\sum }{k_{B}T}\int_{0}^{\sigma }\Phi_{0}(\tilde{\sigma })\;d\tilde{\sigma }$$

where *σ* = −*eϕ*_c_/∑ is the surface charge density, *ϕ*_0_ is the surface potential and *e* is the elementary charge.

The electric potential in solution Φ(*z*) obeys the Poisson–Boltzmann equation [57,58]:

(6) $$\frac{d^{2}\Phi }{dz^{2}}=\frac{2en}{\varepsilon_{W}}\sinh\frac{e\Phi }{k_{B}T}$$

where *n* is the symmetric monovalent salt concentration in bulk and *ε*_W_ is the dielectric constant of the aqueous solution (water). We set the dimensionless electric potential ψ ≡ *e*Φ/*k*_B_*T* and the Poisson–Boltzmann equation can be rewritten as follows:

(7) $$\frac{d^{2}\psi }{dz^{2}}=\ell_{D}{}^{-2}\sinh\psi$$

where *ℓ*_D_ = (*ε*_W_*k*_B_*T*/2*e*^2^*n*)^1/2^ is the Debye screening length. To solve the Poisson–Boltzmann equation, the boundary conditions are given by the following:

(8) $${\frac{d\psi }{dz}|}_{z=0}=\frac{e^{2}\phi_{c}}{\varepsilon_{W}\sum k_{B}T}$$

using Gauss theorem and ψ(*z*→∞) = 0. We can solve the Poisson–Boltzmann equation under these boundary conditions analytically:

(9) $$\Psi =-2\sinh^{-1}\left[\frac{2\pi \ell_{B}\ell_{D}}{\sum }\phi_{c}\right]$$

where Ψ (=*e*Φ_0_/*k*_B_*T*) is the dimensionless surface potential and *ℓ*_B_ is the Bjerrum length defined by *ℓ*_B_ = *e*^2^/(4πε_W_*k*_B_*T*) ≃ 7 Å at room temperature.

By substituting back the surface potential from Equation (9) into Equation (5), we obtain the electrostatic free energy:

(10) $$f_{\mathrm{el}}(\phi_{c})=2\phi_{c}\left[\frac{1-\sqrt{p_{0}^{2}\phi_{c}^{2}+1}}{p_{0}\phi_{c}}+\ln\left(p_{0}\phi_{c}+\sqrt{p_{0}^{2}\phi_{c}^{2}+1}\right)\right]$$

Here, *p*_0_ = 2π*ℓ*_B_*ℓ*_D_/∑ is proportional to the Debye screening length *ℓ*_D_. This charging free energy is also obtained in Ref. [57]. As is the case of the neutral membrane, the chemical potential *μ*_c_ of a charged lipid and the spinodal line are obtained from *μ*_c_ = ∂*f*_tot_/∂*ϕ*_c_ and ∂^2^*f*_tot_/∂*ϕ*_c_^2^ = 0, respectively. They are given by the following:

(11) $$\mu_{c}=\ln\frac{\phi_{c}}{1-\phi_{c}}+\chi (1-2\phi_{c})+2\sinh^{-1}(p_{0}\phi_{c})$$

(12) $$\chi_{\mathrm{sp}\_c}=\frac{1}{2\phi_{c}(1-\phi_{c})}+\frac{p_{0}}{\sqrt{p_{0}^{2}\phi_{c}^{2}+1}}$$

For the strong screening limit (*p*_0_→0), the expression of the spinodal line approaches the neutral case. For the weak screening limit (*p*_0_→∞), the spinodal line can be written as follows:

(13) $$\chi_{\mathrm{sp}\_\mathrm{weak}}=\frac{1}{2\phi_{c}(1-\phi_{c})}+\frac{1}{\phi_{c}}$$

We can obtain the critical point in the case of the weak screening limit from ∂*χ*_sp_weak_/∂*ϕ*_c_ = 0 and the critical point approaches (*ϕ*_cc_, *χ*_cc_) = $\left((3-\sqrt{3})/2,2+\sqrt{3}\right)$ with an increase in *p*_0_.

The thermodynamic potential is also defined by *g*_c_ ≡ *f*_tot_ − *μ*_c_*ϕ*_c_. The coexistence region is determined in the same way as for a neutral membrane. The calculated binodal and spinodal lines are shown at the top of Figure 2a. The solid and dashed lines are the binodal and spinodal lines, respectively. In this calculation, we use *p*_0_ = 6.8, which is consistent with *ℓ*_D_ = 10 Å and *n* = 100 mM. The critical point indicated by the filled circle is located at (*ϕ*_cc_, *χ*_cc_) = (0.628, 3.69). The coexistence region of a neutral membrane is larger than that of a charged membrane. In addition, the critical *χ* value that is inversely proportional to the miscibility temperature is higher in the case of a charged membrane.

These results are consistent with previous experimental reports (Figure 2b) [30,47,49]. The experiments showed that a phase-separated region in the phase diagram of lipid membranes containing unsaturated charged lipids is narrower than that of neutral lipid membranes. As shown in Figure 2a, essentially similar results were obtained in theory. It was also found that experimental and theoretical phase behaviors in the case of salt addition were in qualitative agreement. Based on these reports, further experimental investigations have been reported on the effects of charge on phase separation. Kubsch et al. studied phase behaviors under the asymmetric screening of salts between a charged bilayer [59]. Himeno et al. found that a saturated charged lipid enhances phase separation, which is opposite to the trend for an unsaturated charged lipid [35,49]. Guo et al. reported that the ionization of a charged head group induced the formation of a new phase [60].

## 4. Phase Separation Induced by Chemical Reactions within a Bilayer

**Figure 3:**
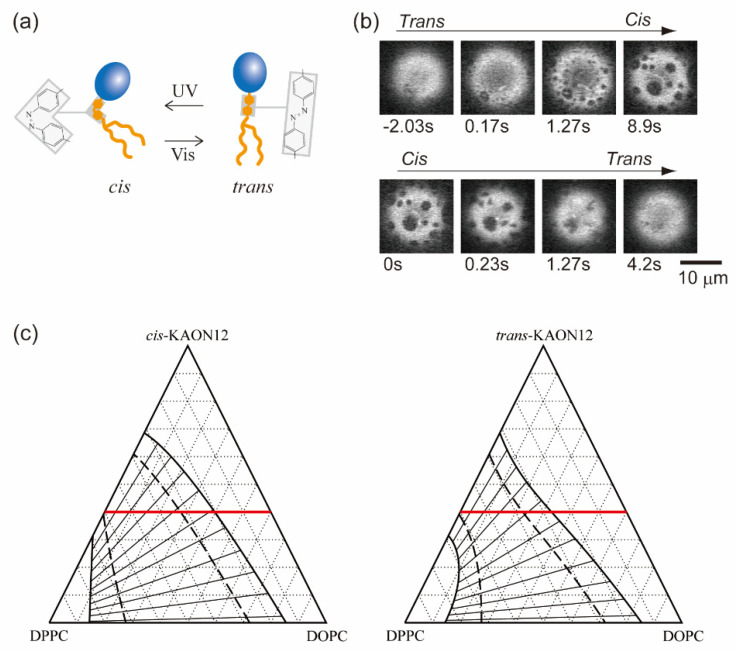
(**a**) Schematics of a synthetic photoresponsive amphiphile (KAON12). (**b**) Chemical reaction-induced reversible phase separation in a model membrane with KAON12. Reproduced from Ref. [62] with permission from The Royal Society of Chemistry. (**c**) Calculated phase diagrams of a ternary system. The heavy solid, dashed and thin solid lines indicate binodal, spinodal and tie lines, respectively. The values of the interaction parameters are as follows: *χ*_us_ = 2.5, *χ*_sp_ = 2.1 and *χ*_up_ = 0 (cis), 0.5 (trans). The red line indicates the mole fraction of 40% KAON12.

Biological membranes are platforms in which various molecules interact and participate in chemical reactions. Not only lipids but also membrane-embedded molecules, such as transmembrane proteins, are laterally sorted within phase-separated membranes according to their affinities for the membrane order [61]. The interaction between molecules within the bilayer contributes to the *χ* parameter of the free energy as in Equation (1), because *χ* is proportional to the energy required to make contact between two different molecules as *χ*~Δ*ε.* Chemical reactions within the membrane may change the molecular interaction energy, which leads to a change in the stability of phase separation. Therefore, in isothermal living systems, chemical reactions of membrane components could trigger the regulation of phase separation. Hamada et al. have developed a simple model system that involves a molecular reaction to control membrane phase separation [62]. A synthetic photoresponsive amphiphile (KAON12) was used, and the molecular conformation (trans or cis) could be switched by using photoirradiation (Figure 3a) [63,64,65]. Cell-sized vesicles were formed from a lipid mixture of dioleoyl phosphatidylcholine (DOPC), dipalmitoyl phosphatidylcholine (DPPC) and cholesterol (Chol) with 40% KAON12. The change in the conformation of KAON12 can produce the reversible switching of membrane phase separation (Figure 3b). Before UV irradiation, the trans-KAON12 membrane surface is homogeneous, without immiscible domains. When UV irradiation causes cisisomerization to occur, the membrane shows phase separation. The two-phase membrane can return to a homogeneous one-phase membrane by transisomerization. This indicates that the miscibility boundary is shifted in the isothermal phase diagram under the photoisomerization of KAON12. Other reports have described the light control of membrane phase separation [66,67,68]. It is noted that photoinduced lipid oxidation also changes membrane properties, including phase separation [69,70].

Next, we describe the physical mechanism of photoinduced phase separation in terms of the Flory–Huggins theory. Here, we extend the Flory–Huggins theory of a binary mixture to discuss phase separation in DOPC/DPPC/Chol/KAON12 membranes. In our experiment, we observed phase behavior by changing the ratio of DOPC/DPPC/Chol, where the proportion of KAON12 was fixed at 40%. Although our system actually includes four components, we consider it to be a DOPC/DPPC/KAON12 ternary component system. As with the binary mixture case, for simplicity we consider saturated lipids to be in a liquid phase by ignoring Chol. The free energy per lipid molecule *f*_usp_ is expressed as follows:

(14) $$f_{\mathrm{usp}}=\phi_{u}\ln\phi_{u}+\phi_{s}\ln\phi_{s}+\phi_{p}\ln\phi_{p}+\chi_{\mathrm{us}}\phi_{u}\phi_{s}+\chi_{\mathrm{up}}\phi_{u}\phi_{p}+\chi_{\mathrm{sp}}\phi_{s}\phi_{p}$$

where *ϕ*_u_, *ϕ*_s_ and *ϕ*_p_ are the mole fractions of DOPC, DPPC and KAON12, respectively. Equation (14) is called the regular solution theory, and it is a basic theory to describe ternary mixtures [71,72]. The mole fractions obey *ϕ*_u_ + *ϕ*_s_ + *ϕ*_p_ = 1 from the conservation of lipid molecules. By substituting back *ϕ*_p_ = 1 − *ϕ*_u_ − *ϕ*_s_ into Equation (14), the free energy can be written as a function of *ϕ*_u_ and *ϕ*_s_. *χ*_us_, *χ*_up_ and *ϕ*_sp_, are the interaction parameters between DOPC and DPPC, DOPC and KAON12, and DPPC and KAON12, respectively. In this model, we ignore the three-body interaction among DOPC, DPPC and KAON12.

We can calculate binodal and spinodal surfaces in the same way as with the Flory–Huggins theory in Section 2. Plotting the free energy in the ($\phi_{u}$, $\phi_{s}$, *f*) space yields a free energy surface, and binodal and spinodal surfaces are calculated in the ($\phi_{u}$, $\phi_{s}$, $\chi_{\mathrm{us}}$, $\chi_{\mathrm{up}}$, $\chi_{\mathrm{sp}}$) space. We here consider phase behavior under the given three kinds of *χ*, and binodal and spinodal surfaces appear as lines. First, the chemical potentials of DOPC and DPPC are given by the following:

(15) $$\begin{array}{l} \mu_{u}=\left(\frac{\partial f_{\mathrm{usp}}}{\partial \phi_{u}}\right)=\ln\frac{\phi_{u}}{1-\phi_{u}-\phi_{s}}+\chi_{\mathrm{us}}\phi_{s}-\chi_{\mathrm{sp}}\phi_{s}+\chi_{\mathrm{up}}(1-2\phi_{u}-\phi_{s})\\ \mu_{s}=\left(\frac{\partial f_{\mathrm{usp}}}{\partial \phi_{s}}\right)=\ln\frac{\phi_{s}}{1-\phi_{u}-\phi_{s}}+\chi_{\mathrm{us}}\phi_{u}-\chi_{\mathrm{up}}\phi_{u}+\chi_{\mathrm{sp}}(1-\phi_{u}-2\phi_{s}) \end{array}$$

The thermodynamic potential is defined by *g*_usp_ ≡ *f*_usp_ − *μ*_u_ *ϕ*_u_ − *μ*_s_ *ϕ*_s_ and the conditions for the coexistence of phases A and B are as *g*_uspA_ = *g*_uspB_, *μ*_uA_ = *μ*_uB_ and *μ*_sA_ = *μ*_sB_. For the given *χ*_us_, *χ*_up_ and *χ*_sp_, the spinodal line in the (*ϕ*_u_, *ϕ*_s_) plane is obtained from the following:

(16) $$\left(\frac{\partial^{2}f_{\mathrm{usp}}}{\partial \phi_{u}^{2}}\right)\left(\frac{\partial^{2}f_{\mathrm{usp}}}{\partial \phi_{s}^{2}}\right)-{\left(\frac{\partial^{2}f_{\mathrm{usp}}}{\partial \phi_{u}\partial \phi_{s}}\right)}^{2}=0$$

Hence, the equation for the spinodal line can be written as follows:

(17) $$\begin{array}{ll} \frac{1}{\phi_{u}\phi_{s}(1-\phi_{u}-\phi_{s})} & -2\left(\frac{\chi_{\mathrm{sp}}}{\phi_{u}}+\frac{\chi_{\mathrm{up}}}{\phi_{s}}+\frac{\chi_{\mathrm{us}}}{1-\phi_{u}-\phi_{s}}\right)\\ & -(\chi_{\mathrm{us}}^{2}+\chi_{\mathrm{up}}^{2}+\chi_{\mathrm{sp}}^{2}-2\chi_{\mathrm{us}}\chi_{\mathrm{up}}-2\chi_{\mathrm{us}}\chi_{\mathrm{sp}}-2\chi_{\mathrm{up}}\chi_{\mathrm{sp}})=0 \end{array}$$

Next, we consider the values of the interaction parameters *χ*_us_, *χ*_up_ and *χ*_sp_. As mentioned in Section 2, the interaction parameter *χ* is inversely proportional to the temperature *T*. In addition, the system becomes homogeneous at *χ* < 2, while phase separation occurs at *χ* > 2. The critical point is at *χ* = 2. Since our observation was conducted at room temperature, the DOPC/DPPC binary mixture undergoes phase separation. Thus, we set *χ*_us_ = 2.5(>2). To obtain the values of *χ*_up_ and *χ*_sp_, we consider the phase behavior of a binary mixture consisting of KAON12 and either DOPC or DPPC. In an experiment on the phase behavior of membranes including photoresponsive cholesterol, phase separation was promoted when the membranes included trans-Chol [66]. On the other hand, our result shows that phase separation proceeds in the case of cis-KAON12. It is natural to consider that this difference is caused by the localization of a photoresponsive molecule. Since Chol prefers to localize in the DPPC-rich phase, we can expect that KAON12 is partitioned in the DOPC-rich phase. This prediction is supported by the fact that phase separation is not observed for DOPC/KAON12 binary membranes at room temperature [64]. Therefore, we assume *χ*_up_ < 2 and *χ*_sp_ > *χ*_up_. In this calculation, we fix *χ*_sp_ = 2.1. Since KAON12 mainly interacts with DOPC, for simplicity, light irradiation only influences the interaction parameter between DOPC and KAON12. Moreover, the values of *χ*_up_ for cis- and trans-KAON12 are taken into account based on the chemical formula of KAON12. Since the structure of cis-KAON12 is bent, cis-KAON12 may disturb the lipid organization; in other words, the value of *χ*_up_ is small. Since trans-KAON12 does not disturb the lipid organization as much as cis-KAON12, *χ*_up_ has a greater value in the case of trans-KAON12.

Using Equations (14), (15) and (17), we calculate the phase diagrams of ternary systems of trans- and cismembranes, as shown in Figure 3c. The interaction parameters are *χ*_us_ = 2.5, *χ*_sp_ = 2.1 and *χ*_up_ = 0 (cis), 0.5 (trans). The heavy solid lines and dashed lines represent binodal and spinodal lines, respectively. The thin solid lines that intersect the binodal and spinodal lines, roughly perpendicularly, denote tie lines. With the mole fraction of KAON12 fixed at 40%, we confirmed the phase behavior experimentally, as shown in Figure 3b. At *ϕ*_p_ = 0.4, the phase-separated region of cis-KAON12 in the calculated phase diagram is larger than that of trans-KAON12. This result is qualitatively consistent with the experimental result from Figure 3b. The theory and experimental results indicate that, when a multi-component membrane is close to the miscibility boundary, chemical reactions of membrane components can trigger the formation of raft domains. Hammond et al. also reported on protein binding in a lipid-induced phase separation using model membranes close to the miscibility boundary [73]. Even if we use other interaction parameters, this result does not change as long as the assumptions about the interaction parameters mentioned above are met. The result from the slope of the tie line indicates that phase separation occurs between the DOPC and KAON12-rich phase and DPPC-rich phase.

## 5. Effect of Membrane Tension on Phase Separation

**Figure 4:**
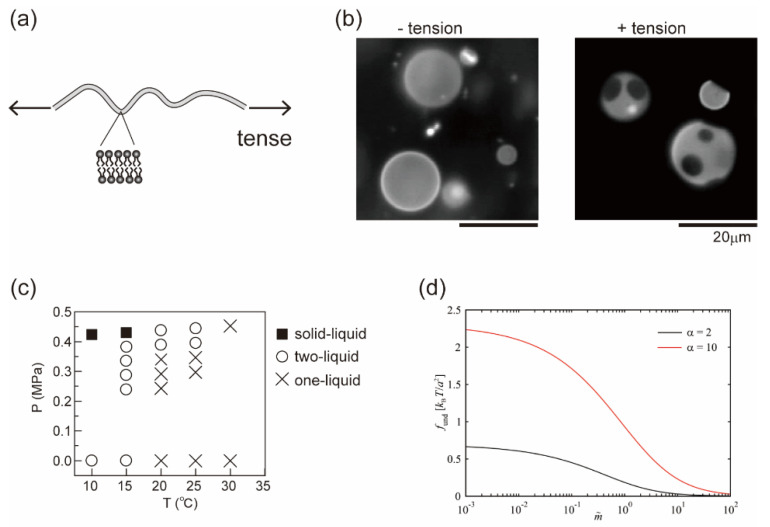
(**a**) Schematic illustration of a tense membrane. (**b**) Typical microscopic images before and after the application of osmotic tension. Reproduced from Ref. [79] with permission from The Royal Society of Chemistry. (**c**) Phase diagram of osmotic pressure and temperature. Reproduced from Ref. [79] with permission from The Royal Society of Chemistry. (**d**) Undulation term of the free energy when the membrane undulation is suppressed. Reprinted with permission from Ref. [87], Copyright 2020 American Chemical Society.

In Section 3 and Section 4, we considered the effects of a molecular property and the reaction of membrane components on lateral phase separation. Here, we focus on an interfacial physical property of the fluid membrane, such as thermal undulation. When membranes are subjected to hypotonic solutions, water passes through the bilayer and the cell volume increases. The membranes then stretch or are placed under tension, where thermal undulation of the membrane interface becomes suppressed (Figure 4a). For semipermeable bilayer membranes, osmotic stress is a fundamental environmental stress. It has been reported that cells respond to hypotonic shock [74,75], an increased membrane tension activates mechanosensitive channels to adjust the osmolality [76,77], and the application of osmotic stress to a cell membrane changes the lipid order to induce cellular signaling [78]. Studies on the osmotic response of lipid membranes are important for a better understanding of the homeostasis of cells. Through the use of model membranes, previous investigations have revealed the effects of osmotic tension on membrane phase organization [60,79,80,81,82,83,84]. Figure 4b shows typical images of a model membrane surface before and after the application of osmotic tension; it was found that hypotonic osmosis induces membrane phase separation [79]. The phase diagram of pressure and temperature showed that osmotic tension increased the miscibility temperature (Figure 4c).

Next, we explain the mechanism of osmotic swelling-induced phase separation in terms of the free energy. The free energy of phase separation can be expressed as follows:

(18) $${∆f}_{\mathrm{tot}}={∆f}_{mix}+{∆f}_{und}$$

where the first term is the Flory–Huggins free energy as in Equation (1) and the second term is the contribution of membrane undulation. ∆ means the energy difference between the phase separation and mixing states as $∆f=f^{\mathrm{separation}}-f^{\mathrm{mix}}$. Theoretical models for describing membrane undulation were introduced by Helfrich [85]; the displacement of undulation is proportional to (*k*_B_*T*/*κ*)^1/2^. When a membrane is stretched due to osmotic swelling, undulation of the membrane is suppressed. To avoid the reduction of undulation entropy, the membrane phase state could be changed. The undulation term was developed by Gordon et al. in [86]:

(19) $${∆f}_{und}≅\frac{\pi k_{B}T}{4a^{2}}\left\{\ln\frac{\alpha +\tilde{m}}{1+\tilde{m}}+2\sqrt{\tilde{m}/\alpha }\;\arctan\frac{1}{\sqrt{\tilde{m}/\alpha }}-2\sqrt{\tilde{m}}\;\arctan\frac{1}{\sqrt{\tilde{m}}}\right\}$$

where *α* = *κ*_B_/*κ*_A_ is the stiffness factor of the membrane (disordered A and ordered B phases with *κ*_A_ < *κ*_B_) and $\tilde{m}$ indicates the degree of the reduction of undulation. They reported that adhesion-induced membrane tension suppressed undulation and induced phase separation [86]. The undulation term in Equation (19) is shown in Figure 4d [87]. Since undulation is suppressed by osmotic pressure or adhesion ($\tilde{m}$ increases), $∆f_{und}$ decreases. This indicates that the loss of entropy to exhibit phase separation, i.e., the formation of rigid domains in a fluid membrane, becomes small when membrane undulation is suppressed. Therefore, tense membranes tend to exhibit phase separation more easily than membranes without tension. Furthermore, based on the results of an image analysis of membrane domains, the line tension of the phase boundary was increased under osmotic pressure [87,88]. Robinson and Dittrich also reported that the compression of vesicles induced domain fusion because of the increase in line tension [89].

Although this theoretical model qualitatively explains the phase separation induced by membrane tension, we have not achieved the understanding of detailed phase behaviors. To describe the phase behavior of tensed membranes, it is important to calculate the total free energy as expressed in Equation (18) and obtain phase diagrams theoretically in future. By proposing such a theory, we expect not only to understand a change in phase behaviors due to membrane tension, but also to show that membrane fluctuations play an important role in phase separation.

## 6. Conclusions

In this review, we have described the physical mechanism of the stability of lipid membrane phase separation under isothermal conditions. A cell-sized model system was adopted to analyze membrane phase behavior, and theoretical models were shown to describe the experimental observations. We considered three typical factors that lead to the regulation of membrane phase separation under cellular isothermal conditions: (i) The presence of charged lipids can suppress phase separation, because their electrostatic contribution changes the binodal and spinodal lines in the phase diagram. (ii) The simple molecular reaction of a membrane component can switch the lateral organization between a homogeneous one-phase and phase separation. The calculated phase diagram explained the on/off switching of phase separation. (iii) Domain formation can be triggered by a change in membrane undulation, which is induced by osmotic swelling. These discussions are based on free energies that describe thermodynamic equilibrium states, and microscopic observations on membrane phase separation under nonequilibrium isothermal conditions have recently been developed [90,91]. These biophysical studies on artificial cell membranes reveal a fundamental law that governs membrane organization. Based on the development of biophysical understanding, fluid mosaic models of the cell membranes have been updated [92]. The possibility of nonraft domain formation was also reported [93]. Further investigations on both cell membranes and model membranes are needed to better understand membrane phase separation under physiological isothermal conditions.

It should be also noted that these studies can contribute to the bottom-up construction of artificial biomimetic systems. Recently, lipid vesicles have been engineered as a synthetic compartment to encapsulate and/or embed functional molecules [94,95]. Not only biochemical factors but also physical factors are of importance to design synthetic cell systems, such as the stability and dynamics of cell-like complex structures [96,97,98]. Membrane surfaces have a significant effect on the behaviors of encapsulated and embedded molecules, since the surface/volume ratio increases in a cell-sized small space [11,99]. The membrane boundary is a fluid, soft interface that exhibits dynamic organization, such as lateral phase separation. The regulation of phase separation could lead to the successful reconstitution of specific molecules within the membrane [99,100], which may relate to the spatial symmetry breaking of a primitive cell model. In addition, it was reported that lateral phase separation can deform the membrane boundary, such as endocytosis-like budding deformation [18,26,101]. Physical understanding of the membrane could provide insight into the design of artificial cells.

## Data Availability

Not applicable.
